# Functional Connectivity Changes in Obsessive–Compulsive Disorder Correspond to Interference Control and Obsessions Severity

**DOI:** 10.3389/fneur.2020.00568

**Published:** 2020-08-20

**Authors:** Iveta Fajnerova, David Gregus, Anna Francova, Eliska Noskova, Jana Koprivova, Pavla Stopkova, Jaroslav Hlinka, Jiri Horacek

**Affiliations:** ^1^National Institute of Mental Health (NIMH), Klecany, Czechia; ^2^Third Faculty of Medicine, Charles University, Prague, Czechia; ^3^Institute of Computer Science, Czech Academy of Sciences, Prague, Czechia

**Keywords:** obsessive–compulsive disorder, functional connectivity, resting state, inhibitory/interference control, Stroop test, obsessions and compulsions, anxiety

## Abstract

**Introduction:** Deficits in neurocognitive mechanisms such as inhibition control and cognitive flexibility have been suggested to mediate the symptoms in obsessive–compulsive disorder (OCD). These mechanisms are proposedly controlled by the “affective” and “executive” orbitofronto-striato-thalamo-cortical (CSTC) circuits with well-documented morphological and functional alterations in OCD that are associated with OCD symptoms. The precuneus region has been suggested in OCD as another key structure associated with the mechanism of “thought–action fusion.” Our study aimed to elucidate the association of the altered functional coupling of the CSTC nodes (and precuneus), the OCD symptoms, and interference control/cognitive flexibility.

**Methods:** In a group of 36 (17 medicated and 19 drug-free) OCD patients and matched healthy volunteers, we tested functional connectivity (FC) within the constituents of the dorsolateral prefrontal cortex “executive” CSTC, the orbitofrontal cortex/anterior cingulate “affective” CSTC, and precuneus. The functional connections showing the strongest effects were subsequently entered as explanatory variables to multiple regression analyses to identify possible associations between observed alterations of functional coupling and cognitive (Stroop test) and clinical measures (obsessions, compulsions, and anxiety level).

**Results:** We observed increased FC (FWE *p* < 0.05 corr.) between CSTC seeds and regions of the parieto-occipital cortex, and between the precuneus and the angular gyrus and dorsolateral prefrontal cortex. Decreased FC was observed within the CSTC loop (caudate nucleus and thalamus) and between the anterior cingulate cortex and the limbic lobe. Linear regression identified a relationship between the altered functional coupling of thalamus with the right somatomotor parietal cortex and the Stroop color–word score. Similar association of thalamus FC has been identified also for obsessions severity. No association was observed for compulsions and anxiety.

**Conclusions:** Our findings demonstrate altered FC in OCD patients with a prevailing increase in FC originating in CSTC regions toward other cortical areas, and a decrease in FC within the constituents of CSTC loops. Moreover, our results support the role of precuneus in OCD. The association of the cognitive and clinical symptoms with the FC between the thalamus and somatomotor cortex indicates that cognitive flexibility and inhibitory control are strongly linked and both mechanisms might contribute to the symptomatology of OCD.

## Introduction

Obsessive–compulsive disorder (OCD) is a chronic neuropsychiatric disorder characterized by recurrent thoughts (obsessions) and by repetitive behavior (compulsions) that is often reported to “neutralize” obsessions and temporarily reduce anxiety (1). Generally, OCD symptoms have been proposed to be mediated by the impaired response inhibition [(2) e.g., (3) meta-analyses by Norman et al. (4)], defined as an ability to suppress pre-potent behavior that is inappropriate or no longer required (5). Affected inhibitory control in OCD, typically demonstrated as difficulty to inhibit irrelevant or distracting information (obsessive thought) and/or behavioral response (e.g., motor rituals) (3). However, alterations proposed in OCD include both inhibitory control (inhibition of motor responses in means of increased impulsivity and/or compulsivity) and the cognitive flexibility processes defined as an ability to shift the focus of attention (6, 7) and/or recognize and handle conflicting information (competition of relevant and irrelevant stimuli) (8). In fact, the inhibitory control could be affected at three consecutive stages of inhibitory control, the early interference control (closely associated with the cognitive flexibility in means of maintenance of conflicting information), the intermediate action restraint/suppression, and the late process of action cancelation (3, 9). Impaired inhibitory control has been reported in OCD patients on all three stages (10), assessed mostly by variants of the Stroop color–word test (SCWT) (7, 11–13), or alternatively by the Go/No-go tasks [e.g., (14)] and the Stop signal tasks [e.g., (15)], measuring interference control, action restraint, and action cancellation, respectively. Even though the impairment in later inhibitory processes of action restraint and action cancelation has not been previously associated with symptom severity (14, 15), it was suggested that the slower reaction times in similar tasks are related to the “not just right experience” reported by the patients (14). Moreover, the fact that motor response is not required to perform compulsion (existence of “pure obsessional” OCD type with primary obsessions and mental form of compulsions, e.g., silently counting or reassurance seeking; (16) points out toward the possibility of primary impairment already at the early stage of inhibitory control (interference control) preceding the perceivable behavioral response. However, the alternative attractor model of OCD by Rolls (17) suggests that impaired interference control reported in OCD results from affected cognitive flexibility associated with the necessary attentional shift (e.g., toward different aspects of the stimuli), suggesting impairment of executive and not inhibitory cognitive processes. However, we argue that these inhibitory and executive cognitive processes cannot be fully separated when using cognitive/behavioral tasks.

Neuroimaging studies have indicated that cognitive flexibility is mainly controlled by the prefrontal cortex (PFC) and frontoparietal attentional network [e.g., (18)]; current reviews suggest the role of fronto-hippocampal communication (19). The interference control and motor response inhibition are both dependent on cortico–striato–thalamo–cortical (CSTC) circuits [for review, see (3, 20)] intensively studied in OCD (21–24). The CSTC loops originate in the PFC and then project to the striatum, from the striatum [via the caudate nucleus (CN)] to the thalamus (THA), and finally from the THA back to the frontal cortex. With special regard to different PFC constituents of these loops, two critical CSTC circuits have been conceptualized as dysregulated in OCD: the “executive” dorsolateral prefrontal cortex (DLPFC)–striatal loop and the “affective” orbitofronto-striatal circuit [orbitofrontal cortex (OFC), anterior cingulate cortex (ACC)], with THA and striatum (CN) belonging to both of them (20). The critical role of CSTC circuits in pathophysiology of OCD has been documented using the fMRI functional connectivity (FC) approach that enables one to investigate how constituents of CSTC networks are integrated and coordinated, and to determine disorder- or symptom-specific disruptions within these networks. Most FC studies have documented that OCD patients exert increased functional coupling within CSTC loops (25–29) and that successful treatment is associated with reduced resting activity within the CSTC loop (30). Particular interest has been recently dedicated to the anterior cingulate cortex region. Structural and functional ACC alterations in OCD patients, including symptom-provocation-induced hyperactivity, has been repeatedly documented in our EEG studies (31, 32). The ACC hyperactivity has been previously linked to performance monitoring (33, 34) and dorsal part of ACC to error monitoring and conflict detection (both possibly contributing to the deficit in cognitive flexibility). Reported functional alterations in this region may thus play a substantial role in the generation of a feeling that “something is not right” preceding compulsions commonly reported by OCD patients (35). Indeed, the hyperconnectivity for tracts originating from ACC and lateral OFC has also been positively associated with the intensity of OCD symptoms (27, 28, 36), and this aberrant connectivity may even represent a specific endophenotype for OCD shared with their first-degree relatives (28). Despite the extensively documented functional alterations of the CSTC loops in OCD, it is not clear how these FC abnormalities (in terms of increased/decreased FC within this network and in outward connections originating from these loops) are associated with specific OCD symptoms. Moreover, studies that would draw a direct link between FC abnormalities in CSTC loop and alterations of cognitive flexibility and inhibitory control processes (suggested as key neurocognitive mechanisms mediating the OCD symptoms (3, 37) are inconclusive, showing both hyper- and hypo-connectivity patterns.

Even though precuneus (PCU) is not a traditional part of the CSTC loops, it has also been suggested as another key structure in OCD. It is densely projecting to PFC and plays a role in so-called thought–action fusion (38, 39) understood as cognitive bias in which an individual believes that specific thoughts and actions are inextricably linked. The fact that OCD patients are particularly prone to this bias (40–42) corresponds to recent structural and functional neuroimaging findings that support the association between OCD symptoms and the alterations reported in PCU such as gray matter reductions (43, 44) and increased precuneal activity (45) corresponding to intensity of symptomatology (46).

However, the relationship between the fundamental processes of inhibitory control and cognitive flexibility suggested to be altered in OCD and the mechanism of thought–action fusion linked to PCU alterations was not directly examined.

Functional alterations within the CSTC loop were repeatedly demonstrated in OCD patients and are still regarded as a central psychopathological mechanism of OCD (29). However, results of resting-state FC studies are often contradictory and they often show no association between FC alterations and symptom severity and/or measured cognitive performance. Our study therefore aimed to answer how altered FC is linked to OCD symptoms and cognitive mechanisms measured by the Stroop test. To this end, firstly, we systematically evaluated FC within regions with structural abnormalities in OCD documented in previous meta-analyses (47, 48) and mega-analyses (21). This set contained the major nodes of two CSTC loops recently identified as crucial for OCD (20). Concretely, we compared patients and control subjects in FC of the “executive” dorsolateral PFC-striatal loop (DLPFC) and the “affective” orbitofronto-striatal circuit (OFC, ACC), THA and striatum (CN) belonging to both of them, and PCU linked to thought–action fusion. Secondly, we tested whether the strength of these OCD-related FC alterations explains the inter-subject variability of cognitive (Stroop score) and clinical symptoms (obsessions and compulsions, and less specific anxiety) in OCD patients. In addition, we also compared medicated and unmedicated OCD patients to evaluate the influence of antidepressants on FC.

Given the previous findings, we hypothesized that FC would be increased in all preselected regions of interest (ROIs). We also expected that FC changes in frontal areas including ACC and DLPFC (responsible for attentional set shift and/or inhibitory control/error monitoring respectively) would correlate with the Stroop test score, while the striatum, thalamus, and precuneus would correlate with clinical symptoms, and none of the FC changes would correlate with the anxiety score.

## Methods

### Participants

In total, 36 in-patients diagnosed with OCD according to ICD-10 (49) and DSM-IV (50) criteria and 36 healthy controls matched for age and sex (see Table 1) were included in the study (all right-handed). Exclusion criteria for all of the subjects involved concurrent severe or chronic medical disease, substance abuse, mental retardation, organic mental disorder, lifetime history of psychosis, mood disorders, severe head injury, and neurosurgery. Healthy controls were also required to have no history of any mental disorder or psychotropic medication use.

**Table 1:** Summary table of demographic characteristics for individual age groups.

		**OCD** ** (*N* = 36)**	**Controls** ** (*N* = 36)**	**Group difference**	
**Demographic**		**Mean score (SD)/** **Sample distribution**		***t*-test /Cramer's** ***V*/Mann–Whitney *U***	***p*-value**
Age		33.26 (8.23)	33.26 (6.74)	*t* = 0.35	0.72
Sex	Males	18	19	*V* = 0.05	0.82
	Females	18	17		
Education (Years)		16.34 (2.84)	17.67 (3.63)	*t* = 1.67	0.10

The severity of obsessions and compulsions in patients was assessed on the day of fMRI session using the Yale–Brown Obsessive–Compulsive Scale [Y-BOCS; (51)] and current anxiety was evaluated by the Hamilton Anxiety Rating Scale [HAM-A, (52)]. The study was approved by the local Ethical Committee and an informed consent was obtained from all of the subjects.

The patients were recruited during the initial phases of the cognitive–behavioral therapy program combining both inpatients and day-care patients. All patients were symptomatic at the time of MRI scanning as evaluated by the Y-BOCS scale, with 1—mild (*n* = 3), 2—moderate (*n* = 21), or 3—severe (*n* = 10) clinical symptoms present. In respect to major symptom dimensions of OCD, recruited patients showed most prevalent dimensions of “contamination/washing” (*n* = 18), “harm/checking” (*n* = 15), and “symmetry/ordering” (*n* = 3).

Nineteen OCD patients were either drug-free or the medication was discontinued at least 5 days prior to measurement. Seventeen patients were medicated with antidepressants (venlafaxin 250 mg, sertraline 50–300 mg, escitalopram 15–40 mg, paroxetine 40 mg, and citalopram 20 mg) and their medication status was stable for at least 4 weeks prior to the study. The patients did not use antipsychotics (occasionally used to augment antidepressant treatment in OCD) at least 5 days prior to scanning. Only short-acting zolpidem was allowed for insomnia. On the day of fMRI, no psychotropic medication was administered before scanning. While all three patients with mild symptoms were in the subgroup of drug-free patients, 8 of 10 patients with severe symptomatology were recruited as medicated; patients with moderate symptomatology were equally distributed to both patients' subgroups (10/11).

### The Stroop Color–Word Test

SCWT is a well-established task (53, 54) for assessing executive functions such as selective attention and interference control. The task was validated in the Czech version (55). It consists of three subtasks/conditions. Firstly, the participant is required to read as many words in the black ink denoting colors as possible. Secondly, the participant is required to name the colors of the displayed stimuli (non-words). The last (interference) task of the test consists of the color–words that are incongruently colored (e.g., the word *red* printed in blue ink). The participant is asked to name the color of the word while trying to inhibit the interference of the automatic tendency of reading the word. Response time in the last condition is slowed because of the competing processing of semantic and visual content of the stimuli. Each card has 100 stimuli to read/name. In the case of finishing before the time limit of 45 s, a participant starts naming from the beginning again. During the task, the participant is corrected if making a mistake. The final scores were calculated as the correct answers achieved in 45 s for each of the subtests: word reading score (W), color naming score (C), and color–word naming score (CW).

### rs-fMRI Data Acquisition

Imaging data were acquired on a 3T Siemens Prisma MRI scanner (Siemens, Erlangen, Germany) equipped with a standard 64-channel head coil. Resting state fMRI (rs-fMRI) was measured with a gradient echo echo-planar sequence (GRE-EPI, TR = 2000 ms, TE = 30 ms, flip angle 70°, bandwidth 2 170 Hz/pixel, without parallel acceleration, FOV = 192 mm × 192 mm, matrix size 64 × 64, voxel size 3 × 3 × 3 mm, each volume with 37 axial slices without an inter-slice gap, a total of 300 volumes). Whole-brain anatomical scans were also acquired using a 3D T1-weighted magnetization-prepared gradient echo sequence (MP-RAGE), consisting of 240 sagittal slices with a resolution of 0.7 × 0.7 × 0.7 mm^3^ (TR/TE/TI = 2400/2.34/1000 ms, FOV = 224 mm), which was used for spatial normalization and anatomical reference.

### Pre-processing of the rs-fMRI Data and FC Analysis and Statistics

FC was analyzed using a seed-driven approach with the latest version (v.17.f) of CONN connectivity software (www.nitrc.org/projects/conn/). The fMRI data were corrected for head movement, together with anatomical scans, and were normalized into standard MNI space and segmented into gray matter, white matter, and CSF tissue classes using SPM12 unified segmentation and normalization procedure (56) and spatially smoothed with a Gaussian kernel (8 mm at full width half-maximum). Physiologic and other spurious sources of noise (the five strongest components of the signal from a region in the cerebrospinal fluid and the region of white matter) were estimated using the implemented component-based method and removed together with movement-related covariates (57). The residual BOLD time course of each voxel was thus obtained from the preprocessed BOLD time series by orthogonalizing it with respect to the tentative confounds [CSF, white matter, realignment parameters, identifiers of outlier scans detected during the outlier identification preprocessing step (corresponding to so-called motion scrubbing), constant and linear BOLD signal trends within each session] and further applying a band-pass filter over a low-frequency window of interest (0.008–0.09 Hz).

The FC analysis proceeded in two steps: first, the relevant functional connections were identified by starting from six key seeds; secondly, the inter-individual differences in strengths of these key connections were analyzed. To establish the overall FC of the network, we used the following bilateral seeds: **OFC**—Orbitofrontal cortex, **DLPFC**—Dorsolateral prefrontal cortex (Superior and Middle frontal gyrus), **ACC**—Anterior cingulate cortex, **CN**—Caudate nucleus, **THA**—Thalamus, and **PCU**—Precuneus. The combined Automated Anatomical Labeling (26 ROIs) and Harvard Oxford Atlas (106 ROIs) provided with the CONN toolbox was used for the FC analysis. For each region of interest (ROI), a representative signal of the ROI was obtained by averaging the residual BOLD time courses across voxels contained in the ROI. The seed-to-voxel connectivity was estimated for the selected seeds by computing Pearson correlation coefficients between the residual BOLD time courses, and further converted to approximately normally distributed *Z* scores using the Fisher transformation. The group effect was evaluated by *between-subject contrast* (equivalent to a two-sample *t*-test). The same analysis was performed for all six preselected ROIs.

**Figure 1:**
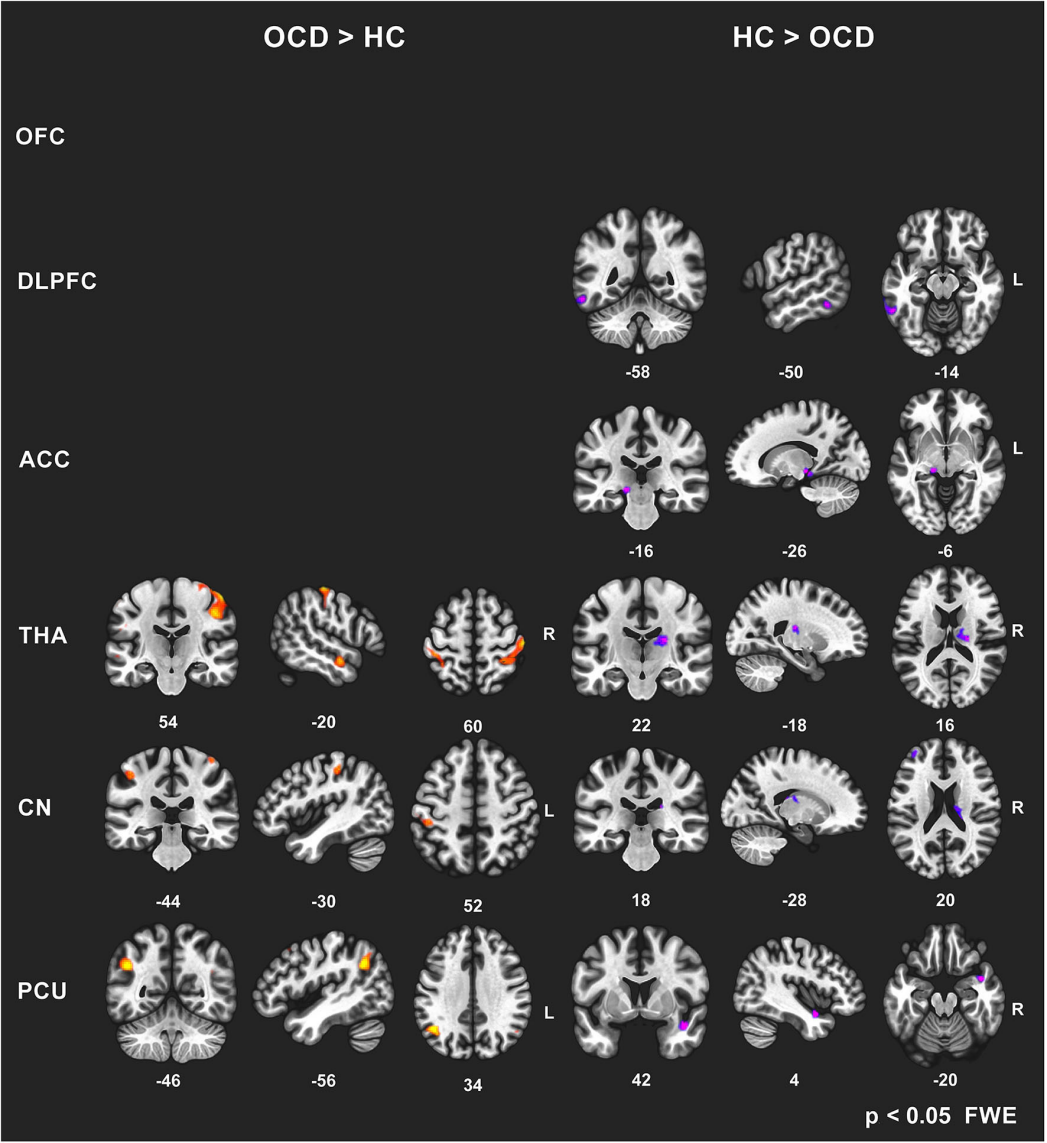
Between-group comparisons of FC effects for individual seeds (brain slice display, FWE corr. *p* level 0.05, with cluster-forming threshold *p* < 0.001). Increased (OCD > HC) and decreased (HC > OCD) functional connectivity in OCD patients for six seeds (OFC—no effect found, DLPFC, ACC, THA, CN, PCU). The clusters obtained using the CONN toolbox are displayed in sagittal, coronal, and axial view in the *x, y, z* coordinates of the local maxima of the strongest cluster. OFC, orbitofrontal cortex; DLPFC, dorsolateral prefrontal cortex; ACC, anterior cingulate cortex; THA, thalamus; CN, caudate nucleus, PCU, precuneus.

To avoid a large number of false positives, for the seed-to-voxel FC mapping, we considered only findings at the conservative cluster family-wise error (FWE) corrected *p* level 0.05 to be significant (with cluster-forming threshold *p* < 0.001). Subsequently, we identified the coordinates of the local maxima of the most significant cluster for each of the six predefined seeds. We identified the anatomical regions (CONN anatomical parcellation) corresponding to these local maxima coordinates. The “slice display” function in the CONN toolbox was used to obtain the presented images of individual brain slices in Figure 1, applying the cluster-FWE corr. *p* level 0.05 with cluster-forming threshold *p* < 0.001 uncorr.).

The aforementioned significant clusters obtained in the seed-to-voxel analyses were exported from the CONN toolbox in a form of cluster masks representing the spatial maps of individual clusters. Similarly, in case of bilateral seeds, the masks covering both areas from the atlas were created. These masks (bilateral seeds and significant clusters) were imported to the CONN toolbox and used in the ROI-to-ROI FC analysis. Finally, Fisher-transformed *Z*-values quantifying the raw connectivity strength during rs-fMRI condition for region pairs (seed-cluster) were used as explanatory variables in the regression analysis in order to evaluate their possible effects on inter-subjects variability observed in cognitive interference measured by the Stroop test (see the statistical analyses below).

Statistica software v13 was applied in additional data analyses and the significance level was set to *p* ≤ 0.05. The Student *t*-test was used to test between-group differences in age and education level; the Cramer's *V* test was applied to test between-group differences in sex distribution. The Student *t*-test was also used to calculate differences in FC of the identified seed-to-target (ROI-to-ROI) pairs in antidepressant-medicated and drug-free patients. The multiple stepwise regression analysis (forward) was used to evaluate whether the functional connections showing the most prominent effects of disease (one for each ROI) explain the variability observed in the cognitive (Stroop CW) and clinical (YBOC-obsession, Y-BOCS-compulsions, HAM-A) scores. We used enter/remove thresholds *F* = 3.84/2.71, approximately corresponding to *p* = 0.05/0.1 as parameters of the stepwise forward variable selection. Pearson correlation analysis was used to calculate potential associations between the cognitive measure (Stroop performance) and individual scores of applied clinical scales measuring severity of obsessions, compulsions and anxiety.

## Results

The group of OCD patients differed significantly (*p* < 0.05) from the matched group of healthy controls in the “color–word naming” subtest score (incongruent word and color). The groups did not differ in the other two subtests that did not involve conflicting information (for details, see Table 2) or in demographic parameters (Table 1).

**Table 2:** Cognitive and clinical measures.

**Measured variable**	**OCD (*N* = 36)**	**Controls (*N* = 33)**	**Group difference**	
	**Mean score (SD)**		***t*-test**	***p***
**Cognitive/stroop color–word test**				
Word list (W)	88.19 (13.75)	93.15 (13.36)	1.516	0.134
Color list (C)	71.97 (12.77)	76.52 (11.12)	1.569	0.121
Color-word list (CW)	43.39 (11.56)	49.24 (9.64)	2.273	0.026^*^
	**Mean score (SD)**		**Score range (min–max)**	
**Clinical/psychiatric scales**				
Y-BOCS obsessions	10.40 (2.85)		5–17	
Y-BOCS compulsions	10.38 (2.80)		3–15	
HAM-A	15.63 (7.81)		3–37	

*W, word list score; C, color list score; CW, color–word (interference) list score; Y-BOCS, Yale–Brown Obsessive–Compulsive Scale; HAM-A, Hamilton Anxiety Rating Scale. The asterisk symbol marks the significance level p < 0.05*.

### Group Differences in rs-fMRI FC

In general, we identified a prevailing increase and less pronounced decrease in FC (5544 vs. 955 voxels over FWE-*p* ≤ 0.05 threshold) in the OCD sample compared to the healthy subjects. The preselected seeds showed differences in between-subject contrast (patients vs. controls, *p* ≤ 0.05 FWE for all reported results, see Figure 1 and Table 3 for positive effects, and Table 4 for negative effects).

**Table 3:** Increased connectivity in the OCD patients compared to HC in the selected ROIs.

**OCD > HC** ** bilat. seed**	***x***	***y***	***z***	**Cluster size**	**Cluster p-FWE**	**Hemi** ** sphere**	**Local maxima**
**OFC**	No effects found						
**DLPFC**	No effects found						
**ACC**	No effects found						
**THA**	**54**	**−20**	**60**	**1421**	**<** **0.0001**	**R**	**Postcentral gyrus**^**+**^
	42	−60	−18	778	<0.0001	R	Fusiform gyrus
	−42	−32	46	774	<0.0001	L	Postcentral gyrus
	−36	−90	−14	767	<0.0001	L	Fusiform gyrus
	52	−4	−16	278	0.0016	R	Middle temporal gyrus
	−52	0	−18	245	0.0034	L	Middle temporal gyrus
	−40	−68	−18	121	0.0870^ns^	L	Fusiform gyrus
**CN**	−44	−30	52	147	0.0259	L	Postcentral gyrus
	−30	−72	12	142	0.0302	L	Lateral occipital cortex
	44	−26	68	126	0.0499	R	Pre- and postcentral gyrus
**PCU**	**−46**	**−56**	**34**	**404**	** <0.0001**	**L**	**Angular gyrus**^**+**^
	50	−64	24	179	0.0111	R	Angular gyrus
	−44	18	54	162	0.0182	L	Middle frontal gyrus

*The table shows the selected bilateral seeds and FC differences observed for comparison OCD patients > healthy controls*.

*x, y, z, coordinates of the local maxima; cluster size, number of voxels; cluster p-FWE, p-value FWE corrected; R or L, right or left hemisphere; local maxima, anatomical regions over the local maxima of effect; OFC, orbitofrontal cortex; DLPFC, dorsolateral prefrontal cortex; ACC, anterior cingulate cortex; THA, thalamus; CN, caudate nucleus; PCU, precuneus. The two results marked in bold represent the seed-target pairs selected for the multiple regression analysis (the target clusters are marked by a plus sign). The cluster p-FWE that is statistically not significant is marked by ns*.

**Table 4:** Decreased connectivity in the OCD patients compared to HC in the selected ROIs.

**HC > OCD** ** bilat. seed**	***x***	***y***	***z***	**No. of voxels**	**Cluster p-FWE**	**Hemi** ** sphere**	**Local maxima**
**OFC**	No effects found						
**DLPFC**	**−58**	**−50**	**−14**	**173**	**0.0165**	**L**	**Inferior temporal gyrus**^**+**^ (temporo-occipital portion)
**ACC**	**−16**	**−26**	**−6**	**145**	**0.0333**	**L**	**Hippocampus**^**+**^
**THA**	22	−18	16	270	0.0019	R	Caudate and thalamus
**CN**	**18**	**−28**	**20**	**279**	**0.0007**	**R**	**Thalamus and putamen**^**+**^
	−36	50	18	127	0.0483	L	Middle frontal gyrus
**PCU**	42	4	−20	134	0.0420	R	Superior temporal gyrus and insula

*The table shows the selected bilateral seeds and FC differences observed for comparison OCD patients < healthy controls*.

*x, y, z, coordinates of the local maxima; cluster size, number of voxels; cluster p-FWE, p-value FWE corrected; R or L, right or left hemisphere; local maxima, anatomical regions over the local maxima of effect; OFC, orbitofrontal cortex; DLPFC, dorsolateral prefrontal cortex; ACC, anterior cingulate cortex; THA, thalamus; CN, caudate nucleus; PCU, precuneus. The three results marked in bold represent the seed-target pairs selected for the multiple regression analysis (the target clusters are marked by a plus sign)*.

However, not all of the preselected seeds (OFC, DLPFC, ACC, CN, THA, PCU) showed a between-group difference in FC. The following efects were observed in the FC of the selected seeds using the *between-subject contrast* (patients vs. controls, p ≤ 0.05 FWE for all reported results) in the seed-to-voxel (whole brain) approach (see Table 3 for positive effects and Table 4 for negative effects). Surprisingly, for the **OFC**, neither positive nor negative FC differences were observed at the level of FWE correction. In the OCD patients (compared to healthy controls), the **DLPFC** seed showed only a decrease in functional coupling with the cluster of the left temporo-occipital and posterior divisions of the inferior and middle temporal gyrus. **ACC** showed only a decrease in FC in the OCD subjects in the hippocampus and the adjacent parahippocampal cortex. In the case of the **thalamus**, highly significant effects (in the means of increased FC in the OCD patients) were found in the four main bilateral clusters consisting of the postcentral gyrus and superior parietal lobule, the fusiform and adjacent occipital cortex, and the lateral temporal cortex covering the superior and middle temporal gyri. Only a decrease in thalamic FC in the OCD patients was observed for inward thalamic voxels and the striatum. In the OCD subjects, the **caudate nucleus** showed an increase in FC in bilateral clusters of post- and precentral gyri, and one cluster located in the left inferior occipital cortex. A decrease in FC was observed in a cluster located in the subcortical gray nuclei (thalamus and putamen) and the left middle frontal gyrus. The **precuneus** showed a strong increase in FC in the OCD patients in three clusters of the bilateral angular gyrus, and the left middle frontal gyrus and less pronounced decrease in one cluster covering the right limbic lobe (superior temporal gyrus, planum temporal, and insular cortex). Given the absence of any effect of medication on FC (see below), we analyzed both medicated and unmedicated patients as one group in further analytical steps. See Figure 1 for the most prominent positive and negative clusters for each of the seeds (apart from OFC).

For each selected seed, the functional connection showing the most prominent effect (largest cluster size) (see effects marked by bold in Tables 3, 4) was used in the subsequent steps of statistical analyses with the following pairs of seed-target regions: bilateral OFC (none); bilateral DLPFC—cluster with local maxima in the Inferior temporal gyrus left; ACC—cluster with local maxima in Hippocampus left; bilateral THA—cluster with local maxima in Postcentral gyrus right; bilateral CN—cluster with local maxima in the right Thalamus; PCU—cluster with local maxima in Angular gyrus left.

### Effect of Medication on FC

Antidepressant-medicated (*n* = 17) and drug-free patients (*n* = 19) showed no differences in functional seed-to-voxel connectivity (for either FWE corrected *p* < 0.05 or uncorrected *p* < 0.001 levels) or in the statistical comparison of FC values extracted for each patient for all of the five seed-target (mask) pairs. The Student *t*-tests calculated for the individual FCs (discriminating the OCD and healthy subjects) did not reveal significant differences between the medicated and unmedicated patients and the results were as follows (seed labels—local maxima of the target cluster): DLPFC—Inferior temporal gyrus left [*t*_(34)_ = −0.598, *p* = 0.554]; ACC—Hippocampus left [*t*_(34)_ = −0.754, *p* = 0.456]; THA—Postcentral gyrus right [*t*_(34)_ = 0.981, *p* = 0.334]; CN—Thalamus right [*t*_(34)_ = 0.056, *p* = 0.956]; PCU—Angular gyrus left [*t*_(34)_ = −0.394, *p* = 0.696].

### Association Between the FC and Cognitive and Clinical Measures

In order to address the *a priori* tested associations between the observed effects in the OCD patients, the strongest FC differences were identified for each of the original ROIs (seeds). As no between-group effect was found for the OFC area, only the functional connections for the five remaining seeds were used as explanatory variables for the multiple regression analyses (DLPFC—Inferior temporal gyrus left, ACC—Hippocampus left, bilateral THA—Postcentral gyrus right, bilateral CN—Thalamus right, PCU—Angular gyrus left). In order to select specific target regions, *Z* Fisher values were extracted from the ROI-to-ROI analysis, representing the seed (masks covering bilateral seeds) and the target mask covering the cluster area [labeled using the local maxima for each of the selected effects (the largest cluster size) obtained in the previous step of the between-group analysis]. The forward stepwise multiple regression analysis (with FC of the seed-target pairs used as explanatory variables) revealed a significant effect for the Stroop CW score, showing that score variability is partially explained by the FC between the thalamus and the cluster with local maxima in the right postcentral gyrus (for details, see Table 5). The FC between thalamus and target cluster (r. postcentral gyrus) was also associated with the severity of obsessions evaluated by Y-BOCS. No association with FC of any of the seed-target pairs was observed for the variables of other clinical symptoms (Y-BOCS compulsions, and HAM-A scores).

**Table 5:** Results of the stepwise multiple regression analysis.

**Response variable**	***F***	***p***	**Adjusted *R*^**2**^**	**Explanatory variable (seed-target pair FC)**	***b***	***p***
Stroop CW score	6.991	0.012^*^	0.146	Right THA–cluster (Postcentral gyrus right)	0.413	0.012
Y-BOCS obsessions	4.142	0.050^*^	0.087	Right THA–cluster (Postcentral gyrus right)	−0.339	0.050
Y-BOCS compulsions	Multiple *R* = 0, no variables entered the equation					
HAM-A	Multiple *R* = 0, no variables entered the equation					

*Forward stepwise multiple regression analyses with the FC of the five strongest seed-target pairs as explanatory variables and cognitive and clinical measures as response variables calculated for the group of OCD patients (N = 36)*.

*CW, color–word (interference) list score; Y-BOCS, Yale-Brown Obsessive–Compulsive Scale; HAM-A, Hamilton Anxiety Rating Scale; b, linear regression estimate Beta*.

*The asterisk symbol marks the significance level p ≤ 0.05*.

No significant correlations (*p* > 0.05) were identified using Pearson correlation in the OCD group between the cognitive measure (Stroop performance in the CW subtest) and the scores of applied clinical scales: Y-BOCS obsessions (*r* = −0.011, *p* = 0.953), Y-BOCS compulsions (*r* = 0.186, *p* = 0.308), and HAM-A (*r* = 0.141, *p* = 0.699). However, the clinical scales were substantially correlated, particularly the severity of compulsions correlated with the severity of obsession (*r* = 0.63, *p* < 0.001) and with evaluated anxiety symptoms (*r* = 0.369, *p* = 0.032).

## Discussion

The main finding of this study is the alterations of FC in the OCD patients with prevailing decrease in cortical constituents of both affective (ACC) and executive (DLPFC) CSTC loops, and both increased and decreased connections from subcortical striatal and thalamic regions and PCU. We also confirmed the altered interference control in the OCD sample. Interestingly, while the alterations observed in the FC of the thalamus are associated with altered interference control measured by the Stroop color–word subtest and severity of obsessions evaluated using the Y-BOCS scale, the FC alterations in other regions of interest did not reveal significant associations neither for interference control nor for clinical symptoms.

### Interference Control (Stroop Test Performance) in the OCD Sample

Our findings of impaired performance in the subtest of the color–word list (but not in the two subtests with no conflicting information involved) in the OCD patients are in line with previous studies reporting altered interference control demonstrated by this SCWT subtest (11, 58). Even though some former studies mentioned neuropsychological slowness in OCD as a dysfunction of fronto-subcortical systems (59), we argue that this would be reflected also in the number of items correctly named during the “word reading” and “color naming” conditions of the SCWT. However, both our results and the results of other studies show equal reading speed and color naming speed for OCD patients and healthy participants (11). Affected performance particularly in the CW subtest suggests that OCD participants may have specific difficulty in maintaining the competing stimuli (color vs. word) and redirecting attention primarily allocated to the semantic meaning of the word. However, we argue that these inhibitory and executive cognitive processes cannot be fully separated, when assessed using the standard cognitive methods. Indeed, the Stroop task examines both processes, by addressing the capacity to process competing information (color vs. text) and control their maintenance (set shifting) and to inhibit irrelevant (yet primary verbal) information provided by the task. Therefore, as both may play crucial roles in the altered task performance, only association of these behavioral measures to FC alterations in OCD patients might potentially clarify their involvement.

### FC Alterations in the OCD Sample

Congruently with the majority of the previous studies on FC in OCD (25–28, 36), our findings documented a prevailing increase (rather than decrease) of functional coupling in the OCD compared with healthy subjects. However, our data document increased FC specifically from bilateral striatal and thalamic regions toward cortical areas of the parietal and occipito-temporal lobe. This finding is fully in line with previous studies aimed at resting FC in OCD (60, 61). Surprisingly, we found only reduced FC originating from the cortical nodes of both executive (DLPFC) and affective (ACC) loops with negative finding in case of OFC, contrary to some of the previous reports showing only an increase in FC within CSTC regions in OCD (29). Nevertheless, this observation is in line with the results of some recent studies both in medicated (60) and in drug-free OCD patients (62), showing decreased FC within the CSTC loop and increased FC originating from central CSTC structures to the regions outside the CSTC, namely, temporal and occipital cortex and postcentral gyrus (60). While the CSTC loop alterations are suggested to be associated with behavioral regulation [e.g., inhibitory control, (60)], the increased connectivity reported outside the CSTC could be related to visuo-spatial and sensory-motor processing. The above reported decrease in DLPFC connectivity with inferior and middle temporal areas corresponds to the decreased PFC connectivity reported by Anticevic et al. (63).

Surprisingly, in contrast to previous studies [for review, see (64)], no evidence of altered connectivity in frontal regions of the OFC (another constituent of CSTC) was found. In a separate analysis, we excluded the idea that this negative finding is mediated by an artifact of medication as medicated and unmedicated patients did not differ in means of FC even at the uncorrected p ≤ 0.001 level. We identified several interpretations for this negative finding. First, the precise location and/or type of alterations reported in this region were heterogeneous across studies, and this could affect the FC results. In concrete, the functional organization of the OFC to medial and lateral divisions of diverse cytoarchitectonic arrays (65, 66) has not been accounted for in our analysis based on recent categorization (20), in which OFC is counted as a single region. To ensure that our negative finding was not affected by the parcellation, we performed a separate *post hoc* analysis addressing separately medial (frontal pole) and lateral (anterior portion of inferior frontal gyrus) division of OFC. As we did not identify any between-group differences for these regions, it is unlikely that our negative finding results from parcellation. However, we cannot exclude the role of technical factors in obtaining high-quality images in this specific region near air/tissue interfaces. Hence, due to the occurrence of susceptibility gradients, the fMRI protocol (GRE-EPI sequence) could compromise the detection of neuronal signals in OFC (67). However, we applied 3T fMRI protocol with 64-channel coil allowing the maximal resolution while covering the whole brain with improved signal/noise ratio. Some specific techniques [such as z-shim compensation; (68)] could achieve better signal detection but only in a selective volume of a single targeted region. Moreover, the strictly conservative correction (FWE) approach applied in the seed-to-voxel based analysis could weaken the chance to detect the between-group differences. We speculate that the OFC FC alterations are not sensitive to the resting state imaging protocol used in our study but that they could be unmasked by functional activation in symptom provocation protocols as in previous studies (69, 70).

Importantly, both increased and reduced FC has been reported by several previous studies for ACC, the constituent of affective CSTC loop (20, 71, 72). The reduced ACC FC with the areas of the limbic/temporal cortex in our sample is in line with recent meta-analysis revealing consistent decreased ACC connectivity with limbic areas (62), temporal cortex, and OFC (61), as well as with the hypoconnectivity within the major brain networks identified by recent meta-analysis (24).

Our findings support the suggested role of the precuneus in OCD. The increased FC of precuneus toward DLPFC and temporo-parieto-occipital junction, specifically the angular gyrus, is congruent with the role of the precuneus in symptom provocation, responsible for awareness of obsessive thoughts and visualization of compulsive actions. It has been suggested (62) that the posterior midline cortex (a part of the default-mode network) is not completely deactivated by the error-signal from the salience network (including ACC). Alternatively, it was also suggested that posterior brain regions such as the posterior cingulate cortex and precuneus may compensate for the dysregulated DLPFC–caudate–thalamus loop and its effect on cognitive flexibility (12, 73).

Together, our findings document the increased resting-state FC from bilateral striatal and thalamic regions (the subcortical nodes shared by both CSTC circuits), decreased FC of ACC and DLPFC to temporo-limbic areas, and more opposed regulatory role of PCU to anterior lateral temporal cortex (negative coupling) and more posterior temporo-parietal region such as angular gyrus (positive coupling).

### Association Between Observed FC Alterations and Cognitive and Clinical Symptoms

Unexpectedly, we did not identify an association between clinical symptoms and cognitive inference and FC originating from striatal and cortical seeds. However, the hyperconnectivity of the thalamus with the somatomotor parietal cortex with local maxima in postcentral gyrus showed association both with obsession severity and with impaired cognitive interference control. This finding is not surprising as these brain areas densely connected by thalamocortical radiations are functionally responsible for speech motor control involving “feedback error detection” in sensory cortices (74, 75). Moreover, this finding corresponds to the impairment of the suggested neural mechanism of conflict monitoring at preexecution stages that activates the subthalamic nucleus, which in turn indirectly inhibits the thalamus (76–78). This would suggest the general role of thalamocortical tracts in cognitive control that plays a role in interference control during performance of the Stroop test. We propose that verbal cognitive processing (reading) of the interfering/distracting information creates a strong semantic attractor for OCD patients that repeatedly fail to shift to an alternative process (17), which is more appropriate (and adaptive) within the context of Stroop test instruction.

In line with a recent study (79), our data suggest the important role of increased coupling between thalamus and the postcentral gyrus in mediating severity of clinical symptoms, documented by convergent findings in obsessions and Stroop interference. Our results suggest that a common neuronal mechanism may underlie both cognitive and clinical scores, in means of interference control (filtering or suppression of irrelevant or intrusive information). As obsessions (intrusive thought) might be explained as representing internal speech processing, the association with the thalamo-parietal FC alterations does not seem random.

We hypothesize that FC of thalamocortical radiations shows association with OCD symptomatology even during the resting state because it is involved in the preexecution stages of feedback error detection (proactive control). On the other side, the FC of cortical regions (constituents of the CSTCs) might be related more specifically to the clinical symptoms when measured during active state such as symptoms provocation. This assumption should be addressed in future studies comparing the strength of association between expression of clinical symptoms and FC in resting and active (provocation) states.

Alternatively, the missing association between FC alterations observed in the other CSTC regions (particularly the frontal lobe ROIs) and clinical symptoms of compulsions (and general anxiety) might be related by the complexity and variability of the compulsions. This variability could be highlighted by the fact that our OCD sample comprised an equal proportion of two OCD phenotypes (18/15)—“contamination/washing” and “harm/checking (aggressive obsessions)”—that might not be directly related to the resting-state individual FC values. Moreover, the strong correlation between the compulsion and obsession severities and moderate correlation with the rated anxiety support the concept of compulsions induced by the intrusive thought and performed to reduce the perceived anxiety.

### Limitations

Some limitations to our study should be noted. Firstly, our clinical sample consisted of drug-free and antidepressant-medicated patients. However, our particular analysis did not detect any difference in FC between these groups (even at the uncorrected *p* ≤ 0.001). Moreover, the effect of medication on fMRI and FC data is limited, if any (80). Secondly, to focus on the functional connections that are altered in the disease, we have used a data-driven definition of the target areas for FC from each of the preselected anatomical regions. This has the advantage of being more specific than purely anatomical definition of target regions, providing a target region that has consistently affected FC to each seed region. However, alternative thresholding of the FC maps could also be considered, leading to a more inclusive or restrictive definition of the target areas. Thirdly, the selection of the most prominent target cluster (i.e., the one with the largest cluster size) for each seed region was used for the multiple regression analyses in order to focus the analysis on the most affected functional connections. Of course, the threshold of five connections is somehow arbitrary and the inclusion of other numbers of connections obtained from the group comparison could, in principle, increase the chance of finding associations between the FC alterations and clinical symptoms; however, particularly including higher numbers of connections could increase the chance of false-positive results. Moreover, our OCD sample consisted of two prevailing OCD phenotypes that could weaken obtained findings. Due to small sample sizes, it was not possible to test potential OCD dimension-specific differences in observed FC alterations, and this should be addressed by future studies. Lastly, to address the distinction between individual stages of the inhibitory control and related cognitive abilities, the future studies should combine several cognitive tasks.

## Conclusion

Our findings demonstrate altered resting-state FC in OCD patients. Specifically, we identified the increased FC from bilateral striatal and thalamic regions (the subcortical nodes shared by both CSTC circuits), decreased FC of ACC and DLPFC to temporo-limbic areas, and more opposed regulatory role of PCU to anterior lateral temporal cortex (negative coupling) and more posterior temporo-parietal region such as angular gyrus (positive coupling). This could be interpreted as a disconnectivity of the traditional CSTC loops in OCD counterbalanced by hyperconnectivity of CSTC regions manifested outwards. In addition, our results support the role of thalamus and its coupling with the somatomotor area of the parietal cortex in OCD, specifically in interference control and/or cognitive flexibility. Moreover, a similar association was identified also for the severity of obsessions, suggesting that suppression (filtering) of irrelevant information might be linked to the altered FC of the thalamus. We suggest that while the affected cognitive control of the conflicting information in Stroop task and severity of obsessions is linked to altered functional state and individual connections of specific brain networks, the complex behavioral clusters of compulsive symptomatology may be caused by more complex or highly individual alterations of brain dynamics that are not captured by individual connections of the studied key hubs of the CSTC network, or that even do not manifest strongly during resting brain state. Future studies should elucidate if the FC in resting and active (symptoms provocation) states differ in the strength of association with behavioral and neurocognitive expression of OCD. Moreover, these studies should separate variable OCD phenotypes (dimensions) and include a combination of methods aimed at inhibitory control and cognitive flexibility.

## Data Availability Statement

The datasets generated for this study are available on request to the corresponding author.

## Ethics Statement

This study procedure involving human participants and all apllied measures were reviewed and approved by the local Ethics committee of the NIMH in Klecany, Czech Republic in accordance with the Declaration of Helsinki. The patients and healthy participants provided their written informed consent to participate in this study prior to participation.

## Author's Note

The well-documented alterations of the orbitofronto-striato-thalamo-cortical (CSTC) circuits are considered a central psychopathological mechanism associated with the symptomatology of the obsessive-compulsive disorder (OCD). The impaired mechanisms of inhibitory control and/or cognitive flexibility have been previously suggested to be mediating the compulsive behavior in OCD, with the involvement of affective and/or executive CSTC loops. Our study aimed to investigate the resting state functional coupling of the CSTCs nodes and to elucidate how the altered functional connectivity pattern is linked to OCD symptoms and cognitive interference control mechanism addressed by the Stroop test. Our findings confirmed altered functional connectivity (FC) pattern in OCD patients (both medicated and unmedicated) with a prevailing increase in FC originating in CSTC regions toward other cortical areas, and a decrease in FC mainly within the constituents of CSTC loops. In addition, our findings support the potential role of precuneus in OCD, a structure previously suggested in so-called thought-action fusion mechanism. Importantly, the associations identified in OCD patients between the functional connectivity of the thalamus and both obsessions severity and cognitive performance indicate that the mechanism of interference control might be linked to specific OCD symptoms, potentially mediated by the mutual constituents of affective and executive CSTC circuits.

## Author Contributions

All authors listed have made a substantial, direct and intellectual contribution to the work, and approved it for publication. Conceptualization: JHo, IF, and PS. Data Acquisition: IF, AF, EN, and PS. Formal Analysis: DG, IF, and JHl. Funding Acquisition: JHo. Methodology: IF, JHo, JK, JHl, PS, DG, and AF. Project Administration: IF, PS, and JHo. Supervision: JHo. Visualization: DG and IF. Writing-Original Draft: IF, JHo, AF, and DG. Writing and Editing: IF, JHo, JHl, DG, AF, EN, PS, and JK.

## Conflict of Interest

The authors declare that the research was conducted in the absence of any commercial or financial relationships that could be construed as a potential conflict of interest.
